# A Miniaturized, Fuel-Free, Self-Propelled, Bio-Inspired Soft Actuator for Copper Ion Removal

**DOI:** 10.3390/mi15101208

**Published:** 2024-09-29

**Authors:** Mohammadreza Chimerad, Pouya Borjian, Pawan Pathak, Jack Fasano, Hyoung J. Cho

**Affiliations:** Department of Mechanical & Aerospace Engineering, College of Engineering & Computer Science, University of Central Florida, Orlando, FL 32816, USA; mo618979@ucf.edu (M.C.); pouya.borjian@ucf.edu (P.B.); pawan.pathak@ucf.edu (P.P.); jack.fasano@ucf.edu (J.F.)

**Keywords:** self-propelled, Marangoni effect, heavy metal ions, water pollution, fuel-free actuator

## Abstract

We present a novel miniaturized, gear-shaped, fuel-free actuator capable of autonomously propelling itself in an aquatic environment to absorb heavy metals, such as copper ions. While hydrogel-based absorbents are promising solutions for cationic pollutant remediation, their stationary nature limits their effectiveness in areas where contaminants are unevenly distributed. To address this, we developed a bio-inspired soft actuator that mimics natural propulsion mechanisms. The Marangoni effect, driven by its inherent chemical properties, demonstrated a self-propelled motion without requiring external fuel. The proof-of-concept actuator generated a plane motion lasting up to 2 h and swept over an area approximately 400 times bigger than its size. By harnessing the chemical and optical properties of the hydrogel, we efficiently removed and quantitatively analyzed copper ions through a colorimetric method. This innovative integration of self-propelled movement and efficient copper ion absorption underscores its potential for advancing miniaturized devices in environmental remediation, paving the way for more active and efficient pollutant removal systems in challenging aquatic environments.

## 1. Introduction

Heavy metal ions have been identified as water and soil pollutants that can potentially endanger public health and cause long-term harm to diverse ecosystems [1]. These non-biodegradable materials possess extended biological half-lives, persisting in aquatic and terrestrial environments [2]. This persistence adversely affects humans and other living organisms [3]. There is a growing public health concern regarding heavy metal contamination, leading to stricter regulations governing the presence of heavy metals in water bodies [4].

Several techniques have been investigated for removing heavy metal ions from aqueous environments to secure safe water resources and human health [5,6]. These methods include ion exchange [7], membrane filtration [8], chemical precipitation [9], and electrochemical techniques [10]. Numerous industries have widely used several of these techniques. However, these procedures exhibit limitations, such as elevated maintenance expenses, substantial electrical power demands, and reduced thermostability [11,12].

Compared to the aforementioned methods, absorption has emerged as a promising candidate for heavy metal removal from the environment due to its unique advantages, including high metal uptake capacity, rapid adsorption kinetics, and potential metal ion selectivity [13,14]. Hydrogels, in particular, have been identified as highly effective absorbent materials with a wide range of applications in heavy metal remediation in wastewater [15,16,17]. They are soft materials created by cross-linking natural or synthetic polymers, forming a three-dimensional mesh structure [16]. Hydrogels possess inherent characteristics, including various functional groups tailored for specific analyte binding, biodegradability, and swellability, making them highly suitable for heavy metal removal [18,19].

Miniaturized self-propelled devices based on a loaded/unloaded power source have evolved as one of the effective instruments for a wide variety of medical and environmental science applications [20,21,22,23,24,25]. Many fuels and propulsion systems based on magnetic, acoustic, optical, and chemical actuation mechanisms have been used to drive such devices [26,27,28]. Among these, using bio-inspired surface-tension-based mechanisms to move the device on the water’s surface has attracted a lot of interest due to their favorable scaling, low toxicity, and high efficiency [29,30,31].

Semi-aquatic arthropods, like rove beetles of the genus Stenus Latreille, are equipped with pygidial glands that emit a complex mix of piperidine and pyridine-derived alkaloids and several terpenes. This secretion composition enables them to skim rapidly and far over the aquatic environment without using their legs. Early observations by Billard and Bruyant noted that these species emit chemicals that act as surfactants, rapidly propelling them forward [32]. This locomotion is driven by Marangoni propulsion, a phenomenon where surface tension gradients created by these emissions allow the beetles to skim swiftly across water surfaces [33]. Self-propelled soft robots have been developed to mimic the locomotion of water striders and move freely on water surfaces [34].

To construct these surface-tension-driven devices, surfactants as fuel must be stored and released in a controlled manner. Numerous studies have focused on this mechanism and developed it to ensure the smooth motion of a swimming device propelled by the Marangoni effect [35,36,37]. While bio-inspired approaches have shown potential, developing self-propelled systems that operate without requiring a surfactant as a stimulus remains a significant challenge.

Despite the proven efficacy of hydrogels in heavy metal removal due to their high absorption capacity and chemical versatility, their static nature limits their application in unevenly distributed contamination scenarios. Additionally, while existing self-propelled devices demonstrate significant promise in medical and environmental applications, the reliance on external energy sources or complex fuel systems complicates their broader deployment. This study addresses these limitations by developing a novel fuel-free, self-propelled soft actuator inspired by the natural motility mechanisms observed in semi-aquatic arthropods, like the Stenus rove beetles. Leveraging Marangoni propulsion, driven by surface tension gradients similar to those used by these beetles, this actuator autonomously navigates water surfaces to target and remove copper ion contaminants effectively. By integrating actuation with remediation capabilities, this bio-inspired device not only overcomes the mobility constraints of traditional hydrogels but also eliminates the need for external power, offering a pioneering approach to environmental remediation.

## 2. Materials and Methods

### 2.1. Materials and Equipment

The following chemicals, materials, and equipment were used: copper (II) nitrate hydrate (heavy metal ions; Sigma Aldrich, St. Louis, MO, USA), 2-acrylamido-2-methylpropane sulfonic acid (AMPS), acrylic acid (AA) (monomers; Sigma Aldrich, St. Louis, MO, USA), polyethylene glycol diacrylate (PEGDA) (crosslinker; Sigma Aldrich, St. Louis, MO, USA), lithium phenyl 2,4,6-trimethyl-benzoyl phosphinate (photoinitiator; Sigma Aldrich, USA), and a laser-powered stereolithography (SLA) 3D printer (Formlabs, Somerville, MA, USA).

### 2.2. Working Principles

The novel fuel-free, self-propelled, bio-inspired actuator was designed to use the dynamic surface properties of hydrophilic–hydrophobic block copolymers and the Marangoni effect to achieve autonomous motion and effective copper ion removal.

**Figure 1:**
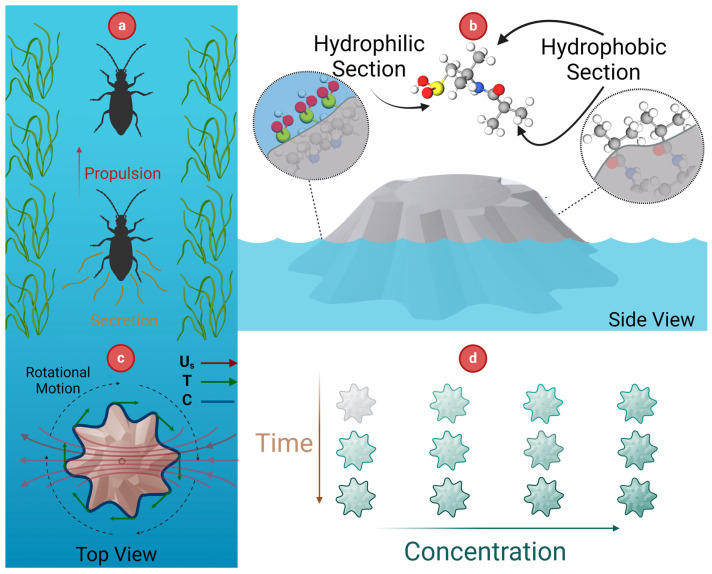
(**a**) Secretion of the surfactant by the beetle, (**b**) rearrangement of the surface configuration in response to the environment, (**c**) theoretical model behind the general plane motion of the gear-shaped actuator, and (**d**) absorption of copper ions indicated by color variations along the concentration and time scale.

The locomotion of rove beetles inspired the operational principle of the actuator. These beetles use the secretion of surfactants to propel themselves forward on the water surface by creating a gradient of surface tension known as the Marangoni effect, as shown in Figure 1a. Theoretically, according to the linearized equation of state for surface tension, changes in surface tension are proportional to changes in surfactant concentration [38]. Furthermore, balancing the tangential viscous stress in the fluid with the tangential force resulting from the gradient of surface tension can be expressed as:

(1) $$\mu \frac{\partial u_{s}}{\partial z}=-\nabla_{s}\gamma$$

where μ, $u_{s}$, and γ represent dynamic viscosity, tangential velocity, and the fluid’s surface tension. Hence, rove beetles applying the surfactant results in a surface tension gradient around the object. Considering the imbalance between the forces, this gradient induces the beetles to start moving and forms a velocity field around the arthropods.

Emulating rove beetles’ biological mechanism, the actuator was fabricated from a chemical structure that undergoes quick surface rearrangement in response to the nature of environmental stimuli [39,40,41,42]. Under dry conditions, the surface of the actuator becomes hydrophobic, whereas it turns hydrophilic upon exposure to water (Figure 1b). This dynamic alteration in surface properties is associated with a change in the contact angle (θ), which, in turn, using Young’s equation, Equation (2), facilitates the generation of the gradient of surface tension [43]. In this equation, $\gamma_{sg}$, $\gamma_{sl}$, and $\gamma_{lv}$ stand for solid/liquid tension, solid/vapor tension, and liquid/vapor interfacial tension, respectively.

(2) $$\gamma_{sg}-\gamma_{sl}=\gamma_{lv}\cos\theta$$

In our device, the integration of Equations (1) and (2) demonstrates that variations in the contact angle are correlated with changes in surface tension, resulting in generating a velocity field around the object. This mechanism parallels the locomotion observed in rove beetles, which is induced by surfactant secretion.

The fabricated actuator benefits from the gear-shaped design, enabling a smooth rotational motion of the object in an aquatic environment. The rotational motion of the actuator influenced by the Marangoni effect can be justified using the circulation concept. It quantifies the tendency of the fluid to induce rotational movement in the actuator.

(3) $$F_{Prop}:\Gamma =\oint U_{s}.dr=\oint U_{s}.Tds$$

Circulation (Γ) can be defined as a line integral of the velocity field $U_{s}$ around the actuator’s perimeter, represented by a simple closed curve C, as presented in Equation (3). This can be expressed in terms of the unit tangent vector *T* and the differential arc length ds along the actuator’s perimeter. In the design depicted in Figure 1c, the changes in the *T* vector and the velocity field are due to the asymmetrical geometry and dynamic reorientation of the actuator’s structure, respectively. This results in a non-zero dot product of $U_{s}.T$ during the actuator’s interval motion. Consequently, as shown in Figure 1c and described by Equation (3), this leads to non-zero circulation and rotational motion of the actuator.

Furthermore, the gear-shaped design provides a higher surface area compared to the other geometries, which could increase the device’s capacity for copper ion absorption, as demonstrated in Figure 1d.

### 2.3. Method of Fabrication

**Figure 2:**
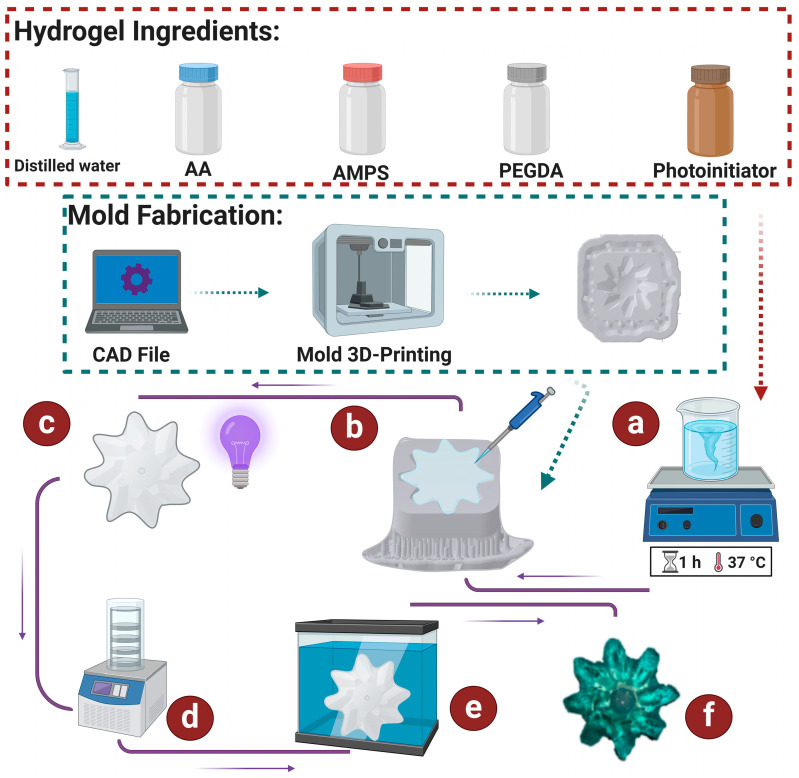
Steps to create and test a gear-shaped self-propelled actuator: (**a**) stirring hydrogel ingredients to form a homogenous solution, (**b**) dripping the prepared solution into a 3D-printed gear-shaped mold, (**c**) using UV irradiation to polymerize the hydrogel, (**d**) freeze-drying the hydrogel to form a porous structure, (**e**) immersing the hydrogel in a copper-ion-containing solution, and (**f**) the actual image of the actuator after copper absorption.

In this study, hydrogel was used to form the actuator’s physical structure. As shown in Figure 2, AA (1 mL, 98%) and AMPS (1.4 mL, 10%) as monomers were copolymerized to form the device’s configuration. Polyethylene glycol diacrylate (PEGDA) (0.04 mL) cross-linked the monomers. The solution contained distilled water as the solvent (2 mL) and lithium phenyl 2,4,6-trimethyl-benzoyl phosphinate (0.009 gr) as the photoinitiator. In contrast, the gear-shaped mold was designed using CAD software (SolidWorks, 2024 version) to create the gear configuration, and the CAD file was transferred to an SLA 3D printer to fabricate the gear mold, as depicted in Figure 2.

As shown in Figure 2a–e, a homogenous AMPS/AA solution was obtained by stirring the combined component solution for 1 h. The stirred solution was then poured into the gear-shaped mold, which was then exposed to UV light to cause the hydrogel to polymerize. The hydrogel was put through a freeze-drying procedure to create a porous structure. Next, the actuator was immersed in a copper-ion-enriched solution, with concentrations ranging from 500 to 3000 ppm, to show its functionality in heavy metal absorption. The actual image of the actuator, which absorbed copper ions, is in Figure 2f.

### 2.4. Material Characterization

The surface and chemical morphology of the AA/AMPS fabricated actuator were examined using a scanning electron microscope (SEM; Carl Zeiss, Jena, Germany). FTIR analysis was conducted to confirm the formation of functional groups in the copolymerized hydrogel. The Fourier-transform infrared spectroscopy (FTIR) spectrum was recorded at room temperature, covering the frequency range of 4000–500 ${cm}^{-1}$ (Shimadzu, Kyoto, Japan).

### 2.5. Locomotion Characterization

The chemical principles underlying the actuator’s locomotion mechanism were evaluated to validate the dynamic changes in the contact angle. To achieve this, contact angle measurements were conducted at room temperature using a contact angle meter (Holmarc, India). Additionally, the associated physical principles were analyzed to correlate the actuator’s average velocity with the hydrogel’s swelling capacity. The velocity was measured from recorded video, and the average velocity was calculated over a 5–10 min interval.

### 2.6. Characterization of Copper Ion Absorption

A series of copper (II) nitrate solutions were prepared at varying concentrations to demonstrate the actuator’s efficacy in heavy metal absorption. These solutions were introduced into a container measuring 32 cm (W) × 48 cm (L) × 8 cm (H). The trajectory and capabilities of the device related to the absorption of copper ions were evaluated using color intensity parameters, including saturation and hue.

## 3. Results and Discussion

### 3.1. Morphological and Chemical Characterization

**Figure 3:**
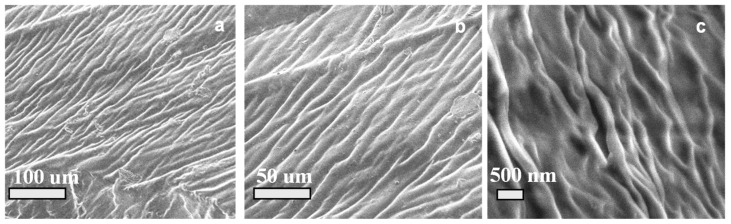
Morphology of the AA/AMPS-based actuator sample at different magnifications with a scale bar of (**a**) 100 μm, (**b**) 50 μm, and (**c**) 500 nm.

Figure 3 illustrates the surface morphology of the fibrous hydrogel that was fabricated, demonstrating the interconnected structure of the self-propelled hydrogel network. The provided SEM images of the actuator revealed a unique surface morphology, showcasing the intricate internal structure at different magnifications.

**Figure 4:**
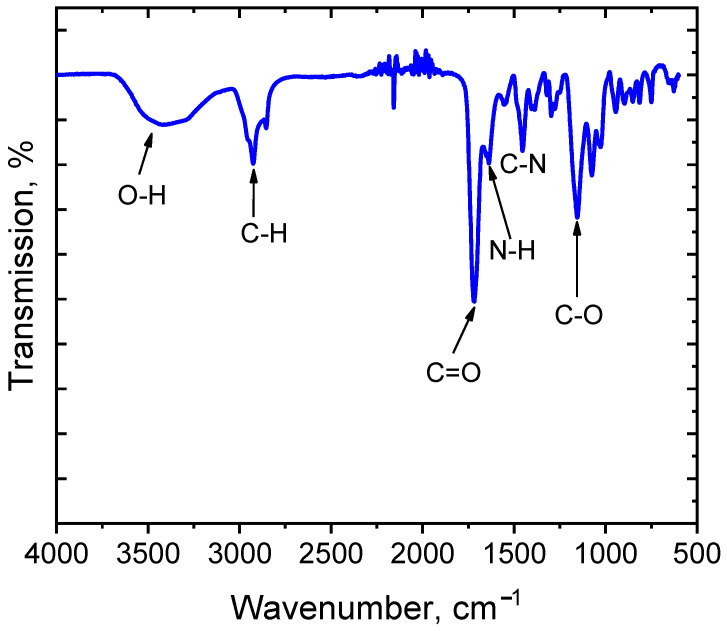
FTIR spectrum of the AA/AMPS-based actuator.

FTIR spectrum analysis confirmed the polymerization process among selected monomers and the exitance of their chemical functional groups, as shown in Figure 4. Peaks at 1357 ${cm}^{-1}$ and 1610 ${cm}^{-1}$ are characteristic stretching vibrations of S=O and N−H associated with the sulfonic acid and amide groups in AMPS. Additionally, peaks at 3300 ${cm}^{-1}$ indicate the elongation of the hydroxyl group in carboxylic acid, indicating AA’s presence in the hydrogel.

### 3.2. Chemical Aspect of Locomotion

**Figure 5:**
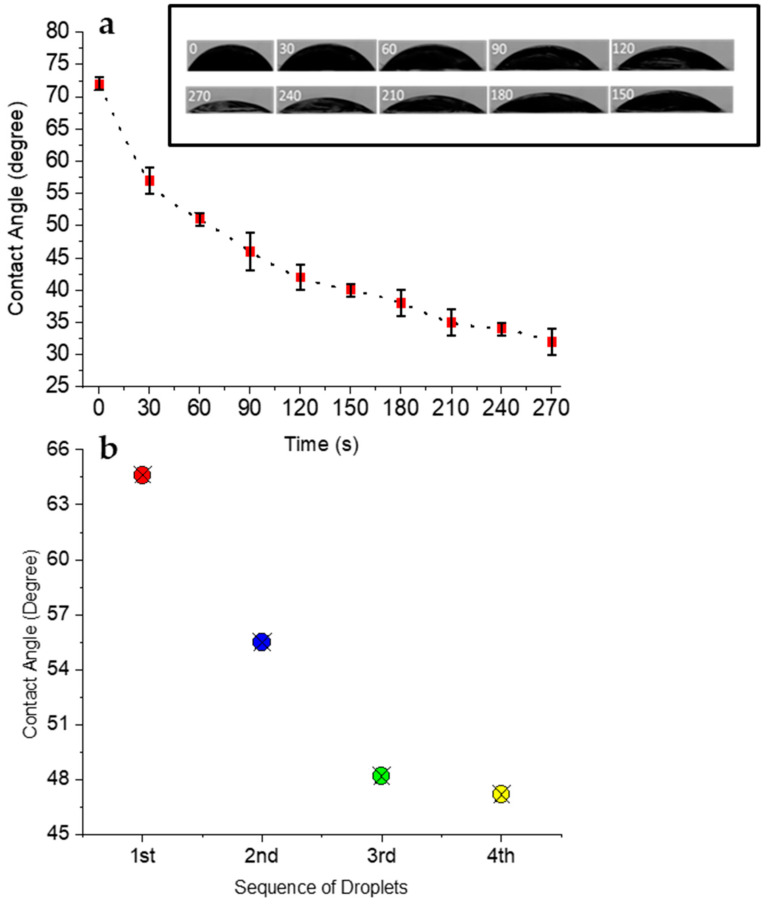
The changes in the contact angle vs. time (**a**) and vs. the addition of sequential droplets at the same spot (**b**).

Figure 5 illustrates the dynamic changes in the contact angle over a short period of time and with sequential droplet addition, indicating corresponding changes in surface tension, as described by Young’s equation. Figure 5a demonstrates the variation in the contact angle versus time to show rapid contact angle changes on the outermost surface of the actuator. The results prove a general decreasing trend in the contact angle in 270 s, indicating a rapid initial decline that gradually stabilizes. Figure 5b shows the contact angles measured at the same spot with the sequential addition of water droplets to highlight a continuous decrease in the contact angle, which is crucial for sustaining the Marangoni propulsion mechanism. Initially, the contact angle was about 64 degrees, and with each new droplet, the contact angle progressively decreased and reached 47 degrees at the fourth one. This steady reduction in the contact angle correlated with a constant decrease in surface tension based on Young’s equation. Hence, the flow in these actuators was generated by alterations in the contact angle, which subsequently changed the surface tension and created a surface tension gradient, resulting in Marangoni propulsion. All the contact angle measurements were performed using the DropSnake method and ImageJ software (Version 1.54k) (Figure 1a,b).

### 3.3. The Physical Aspect of Locomotion

The water-absorbing capacity of the actuator was calculated by the Equation (4) as follows:

(4) $$Swelling\;ratio\;\left(\%\right)=\frac{W_{w}-W_{d}}{W_{d}}\times 100$$

where $W_{w}$ and $W_{d}$ are the weights of the swollen and the initial dried hydrogel, respectively.

**Figure 6:**
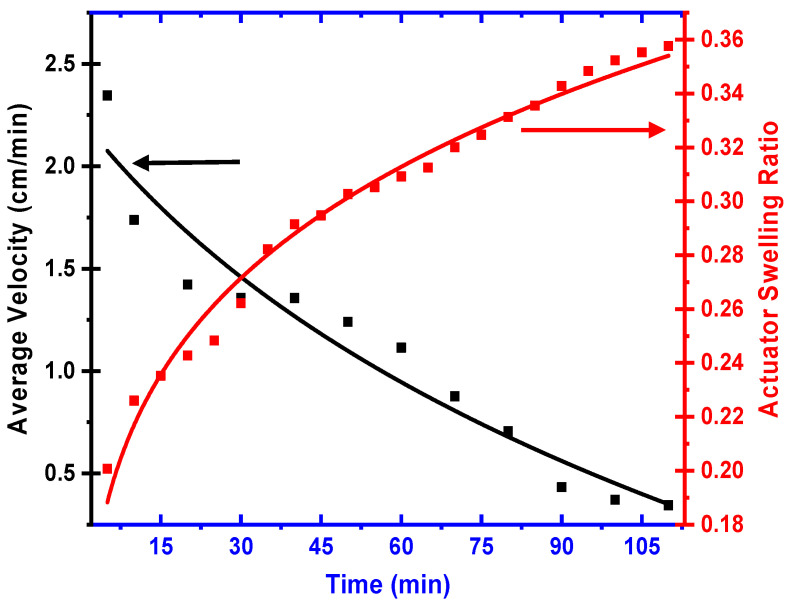
Actuator swelling ratio and average velocity during locomotion time.

The sample was immersed in an adequate copper-enriched solution at 10 min intervals to acquire different swelling ratios. The correlation between the average velocity and the hydrogel’s swelling capacity is shown in Figure 6 to validate the actuator’s locomotion from a physical perspective. As shown in Figure 6, the average velocity of the actuator decreased over time, while the actuator’s swelling ratio increased. This inverse relationship indicates that as the hydrogel actuator absorbs water, it swells, causing a reduction in surface tension, as concluded from Figure 1. Consequently, the Marangoni effect, driven by the surface tension gradient, generates the propulsive force necessary for the actuator’s motion.

### 3.4. Copper Ion Absorption Capacity

The Marangoni effect is crucial in propelling the actuator and enhancing its interaction with copper ions in solution. The hydrogel is composed of anionic functional groups derived from 2-acrylamido-2-methylpropane sulfonic acid (AMPS), featuring sulfonate groups that effectively attract and bind positively charged copper ions through electrostatic interactions. This interaction is dynamically enhanced by the actuator’s continuous movement, driven by surface tension gradients. As the actuator moves, it exposes fresh hydrogel surfaces to the contaminated water, continuously renewing the contact boundary and preventing the saturation that typically reduces the effectiveness of stationary absorption systems. This mobility, coupled with the inherent chemical properties of the hydrogel designed for high metal uptake, enhances the overall absorption efficiency.

**Figure 7:**
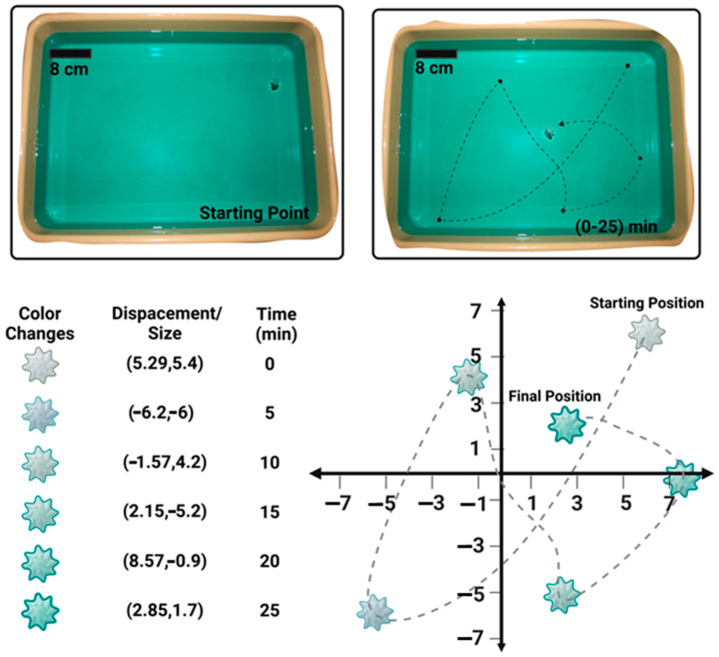
Trajectory of the fabricated actuator immersed in a copper nitrate solution plotted with the normalized scale (displacement/size).

The trajectory of the device and its ability to absorb copper ions are shown in Figure 7, displaying its effectiveness over a 25 min period in an almost $1500\;{cm}^{2}$ area. Furthermore, displacement data were normalized relative to the size of the actuator to provide a scale-independent understanding of its mobility and coverage efficiency. During the trajectory, the actuator had general plane motion, which could be crucial for ensuring thorough exposure to and interaction with a copper-ion-enriched area. This experimental setup validates the actuator’s effective absorption of copper ions over a broad area, confirming its potential as a scalable and efficient solution for heavy metal remediation.

### 3.5. Adsorption Study

**Figure 8:**
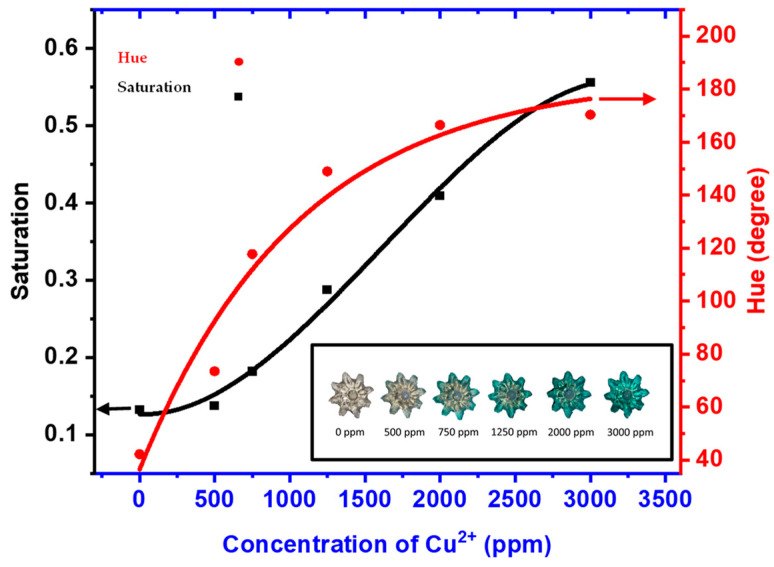
A colorimetric characterization graph of the actuator (inset) shows a color palette for different concentrations of copper ions (500–3000 ppm).

As demonstrated in Figure 8, the AA/AMPS-based actuator was used for the colorimetric analysis of ${Cu}^{2+}$ ions. As the actuator was immersed in the solution, the actuator’s color started changing from the outermost surface area to the middle. The graph illustrates a parabolic and sigmoidal relationship between hue and saturation, respectively, with the concentration of copper (II) ions. As the ${Cu}^{2+}$ ions increased from 0 to 3500 ppm, there was a noticeable transition in saturation from approximately 0.1 to over 0.5, paralleled by a change in hue from 40 to nearly 2000 degrees. This distinct pattern of color variation serves as an effective qualitative indicator of the concentration of ${Cu}^{2+}$ ions.

## 4. Conclusions

In conclusion, this study introduces a novel miniaturized, fuel-free, self-propelled bio-inspired soft actuator applicable to an aquatic environment. It demonstrates its usable features in removing heavy metal ions, particularly copper ions. Using the Marangoni effect for autonomous motion, the gear-shaped hydrogel actuator mimics the rove beetle’s natural propulsion without surfactant loading and unloading.

The actuator is fabricated from a chemical structure that undergoes quick surface rearrangement of functional groups in response to the exposed environment, either air or water. Under dry conditions, the surface of the actuator becomes hydrophobic, whereas it turns hydrophilic upon autonomous exposure to water. This dynamic alteration in surface properties generates a surface tension gradient that enables propulsion.

This innovative approach eliminates the need for external fuel and overcomes the mobility limitations of traditional hydrogels. The actuator demonstrated efficient movement and copper ion absorption. Material characterization through SEM and FTIR confirmed the successful fabrication and functionalization of the hydrogel. The proposed soft actuator’s dynamic contact angle progression enabled Marangoni propulsion, and its swelling behavior kept water absorption and motion in balance. This study provides a unique scalable strategy for removing heavy metals and contaminants from aquatic environments. This fuel-free, self-propelled actuator presents a promising solution for developing autonomous clean-up swarm devices.

## Data Availability

The data supporting this study’s findings are available from the corresponding authors upon reasonable request.
